# Robotic Bronchoscopy for Peripheral Pulmonary Lesion Biopsy: Evidence-Based Review of the Two Platforms

**DOI:** 10.3390/diagnostics11081479

**Published:** 2021-08-15

**Authors:** Abhishek Kumar, Jose D. Caceres, Siddharthan Vaithilingam, Gurshan Sandhu, Nikhil K. Meena

**Affiliations:** 1 Department of Medicine, Division of Pulmonary and Critical Care Medicine, University of Nevada, Las Vegas, NV 89102, USA; 2 Department of Medicine, Division of Pulmonary and Critical Care Medicine, University of Arkansas for Medical Sciences, Little Rock, AR 72205, USA; jcaceres@uams.edu (J.D.C.); svaithilingam@uams.edu (S.V.); nkmeena@uams.edu (N.K.M.); 3 St. George’s University School of Medicine, St. George’s, Grenada; gsandhumed@gmail.com

**Keywords:** robotic bronchoscopy, robot-assisted bronchoscopy, peripheral nodule sampling, lung nodule biopsy, lung cancer diagnosis

## Abstract

Despite many advancements in recent years for the sampling of peripheral pulmonary lesions, the diagnostic yield remains low. Initial excitement about the current electromagnetic navigation platforms has subsided as the real-world data shows a significantly lower diagnostic sensitivity of ~70%. “CT-to-body divergence” has been identified as a major limitation of this modality. In-tandem use of the ultrathin bronchoscope and radial endobronchial ultrasound probe has yielded only comparable results, attributable to the limited peripheral reach, device maneuverability, stability, and distractors like atelectasis. As such, experts have identified three key steps in peripheral nodule sampling—navigation (to the lesion), confirmation (of the correct location), and acquisition (tissue sampling by tools). Robotic bronchoscopy (RB) is a novel innovation that aspires to improve upon these aspects and consequently, achieve a better diagnostic yield. Through this publication, we aim to review the technical aspects, safety, feasibility, and early efficacy data for this new diagnostic modality.

## 1. Introduction

Lung cancer ranks third in the number of newly diagnosed cancers each year, next only to the breast and prostate cancers, but causes the highest number of cancer-related deaths annually in the United States [1]. The advanced stage at diagnosis for the majority of these cases remains a major contributor to this high mortality and hence, the need for early detection at a curable stage. Gould et al. reported that an estimated 1.5 million new lung nodules will be detected annually in the United States [2]. With the recently revised USPSTF lung cancer screening criteria (adults aged 50–80 years with ≥20 pack-years smoking who are currently smoking or have quit within the last 15 years), this number will only increase significantly [3]. The radiographic features are often inadequate in triaging the malignant nodules from the benign ones and biopsy is frequently indicated. 

Lesions located centrally within or adjacent to the airways are amenable to sampling using conventional flexible or convex endobronchial ultrasound bronchoscopy. However, despite many advancements such as the thin/ultrathin bronchoscopes, radial endobronchial ultrasound (rEBUS), and electromagnetic navigation (EMN) systems, the diagnostic sensitivity of the bronchoscopic modalities remains significantly lower than CT-guided transthoracic needle biopsy for peripheral pulmonary nodules (~70% vs. 90%, respectively) [4,5]. That said, this advantage comes with a significantly higher risk of pneumothorax (roughly, 20% with transthoracic needle biopsy vs. 3% with bronchoscopy) [6,7,8,9]. Robotic bronchoscopy (RB) is a novel technology that aims to improve the diagnostic sensitivity of peripheral pulmonary nodule biopsies with fewer procedural complications.

## 2. Materials and Methods

We searched PubMed, Google Scholar, and the reference list of recently published robotic bronchoscopy papers to find all studies since inception pertaining to the performance characteristics of the two available robotic bronchoscopy platforms. Data from the selected papers were extracted by AK and NKM and independently verified by JDC, SV, and GS. Smaller cases/series/trials whose data were included in the larger publications were excluded. Publications detailing technical aspects have been discussed but were not the primary focus of this review. 

## 3. Limitations of Existing Modalities

Conventional flexible bronchoscopes have limited maneuverability in the peripheral airways. With an outer diameter of 4–6 mm and suboptimal single-plane flexibility, they are harder to navigate too far in the periphery. As the airways become smaller distally and tend to often collapse, endobronchial visualization becomes challenging. The newer thin/ultrathin bronchoscopes have a farther reach [10,11]. However, all of them lack a guidance mechanism to navigate successfully to the peripheral targets where both visualization and spatial orientation becomes challenging. Concurrent use of the rEBUS probe has been attempted but did not improve diagnostic sensitivity even in the hands of experienced bronchoscopists [12]. Failure to navigate to the lesion with a lack of control and precision during sampling were identified as the major limitations to success.

CT-to-body divergence (CTBD) is believed to be a major cause of the low diagnostic yield of EMN bronchoscopy platforms. It refers to a difference in the predicted location of the lesion in virtual planning vs. the intra-procedural environment. EMN bronchoscopy requires a pre-procedure thin-slice CT chest in full inspiration. This CT is uploaded to the navigation system which then generates a virtual pathway (failing which, the bronchoscopist manually creates a pathway in the virtual environment). In real-time, patients are variably sedated (most centers use general anesthesia) and often mechanically ventilated for the procedure, with tidal volumes smaller than during the pre-procedure CT scanning. This causes a mismatch between the 3-D virtual and real lesion location. Chen et al. highlighted this phenomenon by demonstrating a 17.6 mm variation in lesion location between end-inspiration and end-expiration [13]. A variation this large could cause the proceduralist to completely miss a <2 cm lesion. For lesions near the diaphragm, diaphragmatic excursions can further worsen this mismatch. Lastly, intra-procedural atelectasis is not present in the pre-procedure CT and consequently not accounted for by the virtual pathway, adding more uncertainty to the intra-procedural lesion localization. Essentially, the navigation success as per the EMN system sometimes may not be true, resulting in failure to obtain a representative tissue sample.

Confirmation of successful navigation has been another limitation. Adding rEBUS to confirm lesion localization was tried but failed to improve the diagnostic yield, perhaps due to the intraprocedural atelectasis in the vicinity of the target lesion. The development of atelectasis in sedated patients has been well documented. Given that atelectasis has a similar ultrasound appearance to the target lesion, rEBUS image-based confirmation may lead to a “false-positive confirmation”, resulting in inadequate or nondiagnostic sampling. The recently published i-LOCATE study highlights this aspect, wherein 89% of the patients undergoing advanced diagnostic bronchoscopy under general anesthesia developed atelectasis in at least one dependent segment. Duration of general anesthesia and body-mass index were reported as independent variables abetting atelectasis in this study. In effect, intra-procedural atelectasis might adversely impact both navigation and confirmation accuracy [14]. Because the EMN systems “assume” a target location based on the virtual planning and are impacted by the CTBD, their lesion confirmation could potentially be just as misleading. To offset this deficit, using cone-beam CT (CBCT) to confirm the biopsy tool placement in the lesion (called the “tool-in-lesion sign”) has been studied. A randomized controlled trial published by Ost et al. in 2008 compared conventional and CT-guided bronchoscopy [15]. The study expectedly reported better diagnostic yield with CT confirmation of the tool-in-lesion, but simultaneously acknowledged that the conventional bronchoscopes available at the time were too large in diameter for peripheral maneuverability and course correction even if they were visibly missing the lesion. Similar limitations in maneuverability were observed for the available biopsy tools as well. A decade later, in 2018, Casal et al. published a small pilot study of peripheral lung nodule biopsy using thin/ultrathin bronchoscopes in 20 subjects [16]. They used rEBUS to confirm navigation success (defined as successfully visualizing the concentric or eccentric lesion image), followed by CBCT to confirm that the rEBUS probe truly made contact with the lesion. This study reported improved navigation and diagnostic yield by 25% and 20%, respectively, highlighting the importance of lesion confirmation. 

A biopsy instrument’s position, maneuverability, and stability are crucial to precise sampling despite successful navigation. Whether the lesion visualization is concentric or eccentric with the rEBUS probe matters—a concentric rEBUS view increases the odds of the biopsy tool being within the lesion and hence, a better yield [17]. As we reach the narrower distal airways, the biopsy tool’s angulation and maneuverability become harder for the eccentrically located lesions. The conventionally available biopsy tools are also relatively inflexible and difficult to get past the acute bend of the bronchoscopes in distal airways. Aggressively “pushing tools out” sometimes causes scope damage. For better confirmation of the “tool-in-lesion” (real-time confirmation of the biopsy tool in the target lesion), the cone-beam CT (CBCT) scan has been tried. CBCT is a mobile CT mounted on a C-arm that has shown potential in real-time confirmation of the tool-in-lesion sign, most importantly, differentiation between a true solid lesion vs. atelectasis. As previously stated, Ost et al. demonstrated improved diagnostic yield with CT confirmation of the tool-in-lesion [15]. Electromagnetic navigation systems have their own limitations. Aside from the CTBD negatively impacting both navigation and lesion confirmation, the lack of an “air bronchus sign” (presence of an airway leading to the target lesion) has been associated with a lower diagnostic yield [18]. Furthermore, the sheath sometimes moves or gets dislodged after lesion localization just before the biopsy, i.e., their instability often contributes to sampling inaccuracy. Overall, all the above-mentioned limitations decrease the diagnostic sensitivity of the available bronchoscopic modalities for biopsy of the peripheral lung nodules. Newer technologies such as robotic bronchoscopy aim to improve on all three key aspects, namely navigation, confirmation, and acquisition success, for peripheral lesion sampling. 

### 3.1. Robotic Bronchoscopy Platforms

#### 3.1.1. Monarch^TM^ Robotic System

**Figure 1:**
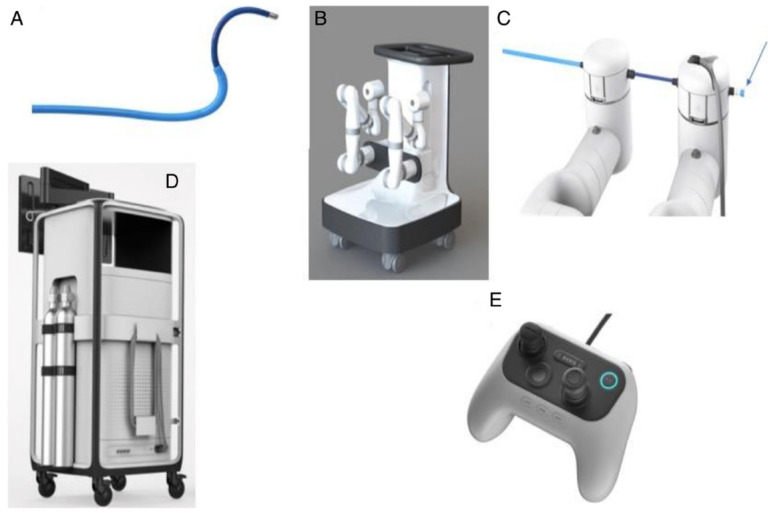
The Monarch^TM^ Robotic System (Auris Robotics). Reproduced with permission from Dr. Septimiu Murgu, and under Open Access terms of the creative commons license (http://creativecommons.org/licenses/by/4.0), accessed on 29 July 2021. The system consists of (**A**) an outer sheath-inner scope bronchoscope assembly, (**B**) a cart with two robotic arms, (**C**) a scope-to-arm attachment with provision for pushing saline/air, suctioning and tooling (arrow), (**D**) a tower with a display monitor, and (**E**) the controller.

The Monarch^TM^ Robotic Endoscopy System (Auris Robotics, Redwood City, CA, USA) was the first robotic endoscopy platform to be approved by the US Food and Drug Administration in March 2018. Much like the preexisting EMN systems, the Monarch^TM^ system uses an electromagnetic field generator and reference sensors. The system (Figure 1) is comprised of a robotic bronchoscope system capable of articulation in pitch (vertical) and/or yaw (horizontal) or both, two robotic arms that drive the scope, a handheld controller, and a tower with two computers running the robot and a display showing both the EMN and real-time camera views. The bronchoscope system has an outer sheath and an inner video bronchoscope, and with telescoping capability, imparts enhanced endoscopic control and reach in the peripheral airways. It allows for 4-way steering with a 180-degree scope tip deflection in any direction. The outer diameter of the sheath is 6.0 mm and the scope is 4.2 mm. The working channel’s diameter is 2.1 mm. Auris manufactures a flexible needle (currently recalled for revision) designed for the Monarch^TM^. However, any of the available tools currently being used through the 2 mm working channel fit in too and can be used as needed. 

As a part of the pre-procedure planning, a thin-cut (1 mm or less) CT scan of the chest is performed, based on which a 3-dimensional virtual lung reconstruction is generated. The planning software then automatically maps pathways (failing which, manual planning can be done) to the target, which are uploaded to the robot system. The next step is the registration, much like the current EMN systems, to link the virtual planning with the patient’s airway anatomy. The proceduralist thereafter navigates to the target of interest using the controller console. The system is designed to provide live feedback on the location and distance to the nodule as well as continuous direct visualization during navigation and sampling, attributes that potentially improve tissue acquisition. 

It is worth mentioning that the manufactures of the current EMN bronchoscopy platforms do not recommend their use in patients with cardiac implantable electronic devices (CIED) like cardioverter-defibrillator or pacemakers. With that said, a few investigators have reported acceptable safety profiles in smaller trials, acknowledging the need for larger validation studies [19]. In our previous publication, we assessed the risks and management of electromagnetic interference for patients with CIEDs in various IP procedures, including EMN platforms [20]. Most patients seem to tolerate the procedure well, but a cardiology consultation with pre- and post-procedure device interrogation is advisable. Other measures such as using the magnet or reprogramming may be needed as warranted by the cardiologist. A recent expert review proposed another interesting approach in these patients with using just the bronchoscopy system without turning the EMN guidance on, taking advantage of the enhanced reach and stability [21].

#### 3.1.2. Ion^TM^ Robotic System

**Figure 2:**
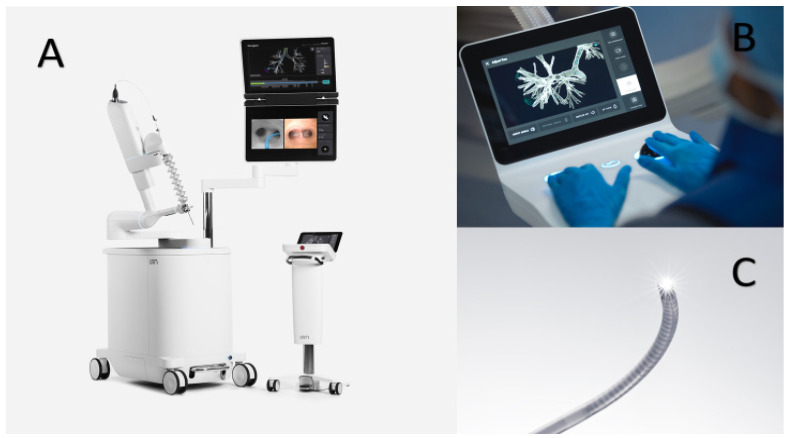
The Ion^TM^ Robotic System (Intuitive Surgical). Images © (2021) Intuitive Surgical, Inc. The system consists of (**A**) the main system cart with articulating catheter-inner vision probe bronchoscope assembly and attached monitor plus the controller console, (**B**) the trackball and scroll-wheel controller, and (**C**) the fully articulating catheter with the removable vision probe.

The Ion^TM^ Robotic Endoluminal System (Intuitive Surgical, Sunnyvale, CA, USA) was approved by the US Food and Drug Administration in February 2019. It uses a shape-sensing technology instead of electromagnetic navigation. The system (Figure 2) comprises the flexible robotic catheter containing shape-sensing fibers through its entire length, a removable video scope that sits in the catheter during navigation providing direct visualization, planning station, robot cart with display screens, and a controller with a trackball and a scroll wheel. The outer diameter of the catheter is 3.5 mm with a working channel of 2.0 mm, capable of accommodating any tool compatible with the existing 2.0 mm channel bronchoscopes. The Ion^TM^ has its own proprietary flexible biopsy needle (Flexision) with 3 mm stroke length, available in 19 G, 21 G, and 23 G sizes. The articulating catheter can turn 180-degree in any direction. In contrast with the Monarch^TM^ system, the Ion^TM^ video scope must be removed from the catheter after satisfactory navigation to accommodate the sampling tools. Thus, direct visualization while sampling is not available.

Similar to the other navigation systems, a thin-cut pre-procedure CT chest is performed based on which the Ion^TM^ PlanPoint^TM^ software creates a 3-dimensional virtual airway reconstruction and automatically (done manually if needed) identifies possible pathways. This is followed by the registration akin to the EMN platform to connect the virtual planning and real-time navigation, with the difference being that Ion^TM^ uses the shape-sensing catheter instead of points in the electromagnetic field. The system provides live inputs on the direction of and distance to the target, as well as the distance from the nearest pleura. The system software also recommends the optimal fluoroscopic angle with the best planar view of needle strokes to avoid pleural puncture. Once successful navigation is accomplished, the catheter is locked in position and the vision probe removed to accommodate the biopsy tools. 

During sampling, both the systems use modalities such as fluoroscopy, radial EBUS, and cone-beam CT when available to confirm navigation success and “tool-in-lesion sign” to improve upon the tissue acquisition. Their advantages and pitfalls are discussed in the discussion section. Table 1 lists a comparison of the technical aspects of both the systems.

### 3.2. Current Evidence for Robotic Platforms

#### 3.2.1. Monarch^TM^ Robotic System

The REACH (Robotic Endoscopic Airway Challenge) Assessment [22] was a cadaveric study using the Monarch^TM^ system. In this study, Chen et al. compared the accessibility of the peripheral airways in two human cadavers using the conventional thin bronchoscope (4.2 mm scope; 2.0 mm working channel) vs. the Monarch^TM^ robotic bronchoscope (6.0 mm outer sheath; 4.2 mm inner scope; 2.1 mm working channel). All segmental bronchi (RB1-10 and LB1-10) were accessed using both modalities. The robotic bronchoscope was able to reach farther in all the segments both by the insertion depth as well as airway generation count. The difference was particularly impressive in the segments with increased angulation, such as RB1 and LB 1+ 2 (mean generation count 8 vs. 3.5, respectively) and LB1D2 (mean generation count 8 vs. 4.5).

The ACCESS study [23] was the first accuracy trial using the Monarch^TM^ system. Artificial target lesions (10–30 mm in axial diameter; mean size 20.4 mm; mean distance from pleura 1.6 cm) were implanted in eight human cadaveric models that were intubated and mechanically ventilated for the procedure. Eight bronchoscopists received prior training in performing navigation and biopsies on the new platform, but no assistance was provided during bronchoscopy. Direct endoscopic visualization, EMN guidance, fluoroscopy, and radial EBUS were all used for each procedure. The proprietary 24-G Auris Health flexible needle (forceps biopsies were optional) was used for biopsies. Samples were considered diagnostic when the pigmented mica powder used to create the artificial targets was obtained. The overall diagnostic yield was excellent at 97% for these artificially implanted lesions in the cadavers. No statistical difference in the diagnostic yield was found between >2 vs. <2 cm lesions, eccentric vs. concentric radial EBUS views, or with respect to the distance from the pleura. No airway trauma was reported following a thorough post-biopsy inspection during scope removal.

Rojas-Solano et al. conducted the first safety and feasibility study of the Monarch^TM^ system in humans [24]. This was a single-center prospective case series that enrolled 15 of the 17 patients screened to undergo robotic bronchoscopy. The primary endpoint was the safety, i.e., the complication rate of the device or procedure-related serious adverse events. The secondary endpoint was the technical success, i.e., the ability of the Monarch^TM^ system to complete the intended procedure. The direct visualization capability of the system was also assessed during the deployment of biopsy tools and inspection of the bronchial tree during the procedure. Twelve of these subjects had peripheral lesions and three had central lesions. The median lesion size was 2.6 cm (1–6.3 cm) and the median distance from the pleura was 0.6 cm. Notably, the presence of bronchus sign was one of the inclusion criteria (other criteria being age ≥ 18 years, CT scan within 30 days, and the ability to provide informed consent). Moreover, rEBUS and EMN support was not provided during the procedure, making the robotic system the sole guiding modality for peripheral navigation. All procedures were performed by two experienced bronchoscopists who underwent ≥6 h of pre-procedure training on the Monarch^TM^ system. Patients were intubated and mechanically ventilated under general anesthesia for the procedure. All patients underwent two prespecified follow-up visits 2 and 7 days after the procedure. Adverse events and symptoms were recorded on both these visits, and the final pathology result was recorded on the 7-day visit. For the primary endpoint, no significant adverse events (including pneumothorax and significant bleeding) were reported. Three patients reported minor unrelated complications (fever 4 days post-procedure [1], anesthesia-related nausea/vomiting [1], and back pain found to be due to the paravertebral muscle contracture [1]). The secondary endpoint was achieved with successful tissue acquisition in 14/15 or 93% of the patients. Biopsy tools could be visualized in all the cases during sampling. Conventional flexible bronchoscopy was required in 1/15 patients for a right upper lobe lesion that was found malignant. Another patient required subsequent surgical biopsy to confirm malignancy in a nondiagnostic bronchoscopy biopsy.

The first large multicenter experience with the Monarch^TM^ system was published by Chaddha et al. as a retrospective study [25]. Four centers enrolled 165 consecutive patients (with 167 lesions) who underwent guided bronchoscopy for both suspected malignant and nonmalignant (mycobacterial, fungal, etc.) lesions. Two cases underwent a biopsy of two lesions each during the same procedure. If lesions were identified on the pre-procedure conventional white-light bronchoscopy, they were excluded from the analysis. All procedures were carried out under general anesthesia while patients were intubated and mechanically ventilated. Navigation was performed using direct visualization as well as the system-generated virtual pathway in tandem. Radial EBUS was used before the biopsy to confirm lesion localization. The mean lesion size was 25.0 ± 15.0 mm, with 71.3% lesions ≤30 mm and 70.7% of the lesions located in the peripheral 1/3rd of the lung. Roughly 1/3rd had an associated bronchus sign on the CT and an almost equal proportion were the solid nodules. Successful navigation was achieved for 148/167 (88.6%) lesions and visual confirmation by rEBUS accomplished in 141/167 (84.4%; concentric = 57.5%; eccentric = 42.5%) lesions. Sample acquisition was successful in 161/165 patients (97.6%; failure to navigate - 3; software/equipment failure - 1). A needle was used in all the cases and forceps in 96% of cases. The mean navigation and procedure times were 17.8 ± 19.1 and 58.6 ± 31.4 min, respectively. All patients were scheduled for follow-up within the 185 ± 55 days timeline post-procedure. The final diagnostic sensitivity was reported as 69.1 vs. 77%, considering only inflammation reported on pathology report as non-diagnostic vs. diagnostic, respectively. The yield varied greatly based on the pattern of rEBUS visualization of the lesions (81.5, 71.7, and 26.9% for concentric, eccentric, and no visualization, respectively). Lesions with bronchus sign vs. without had a higher yield (78.3 vs. 54.1%, respectively). Diagnostic yield was no different for solid vs. semisolid and central vs. peripheral lesions. In terms of complications, pneumothorax occurred in 6 (3.6%) cases, 4 (2.4%) of which required tube thoracostomy. Major bleeding requiring cold saline instillation was reported in 4 (2.4%) cases. None of the patients required blood products, bronchial blockers, or further therapeutic interventions. No other procedure complications, respiratory failure, or death were reported. The study concluded that robotic bronchoscopy is safe and feasible, and should be offered to patients requiring peripheral lesion sampling, especially in combination with EBUS-TBNA for appropriate indications.

The BENEFIT study is the latest reported trial for the Monarch^TM^ system [26]. Chen et al. performed the first prospective multicenter pilot and feasibility study using this platform. They enrolled 55 patients with peripheral pulmonary lesions (without mediastinal/hilar disease) across five centers to undergo robotic bronchoscopy-guided biopsy. The primary endpoints were successful lesion localization and the incidence of procedure-related adverse events. All procedures were performed under general anesthesia and so, patients were intubated and mechanically ventilated. Navigation was accomplished using direct visualization, electromagnetic navigation, and fluoroscopy. Radial EBUS was then used to confirm lesion localization, following which transbronchial needle aspiration (TBNA) was performed. Rapid on-site evaluation (ROSE) was performed for all procedures. When ROSE was diagnostic, the procedure was terminated after TBNA alone. When nondiagnostic, transbronchial biopsy was performed. If both had a negative ROSE, the study design allowed additional crossover conventional guided bronchoscopy biopsies (recorded separately) as deemed necessary by the investigators. The lesions measured 1–5 cm in diameter. The median size was 23 mm with an IQR of 15–29 mm, and 78% of the lesions were ≤30 mm. Bronchus sign was seen in 32/54 (59.2%) of the lesions. Of the 55 subjects enrolled, one withdrew consent leaving 54 subjects in the final analysis. Successful lesion localization was confirmed by rEBUS visualization in 51/53 (96.2%; concentric view = 60.8%) cases that underwent rEBUS confirmation. The median time to lesion confirmation was 13 min (IQR 10–24 min) and the median procedure time was 51 min (IQR 44–54 min). The diagnosis was made in 40/51 cases (sensitivity 74.1%; 95% CI 61–84%). Of those testing positive, 33/40 were malignant. Crossover procedures were performed in 5 patients with nondiagnostic ROSE. One additional diagnosis was made using the thin bronchoscopy plus rEBUS followed by TBNA. Another case that underwent a crossover procedure yielded negative biopsy results but had a positive final cytologic report from a robotic bronchoscopy sample. The remaining three cases remained nondiagnostic despite both the robotic and crossover procedures. Assessing for complications, 2/54 (3.7%) of cases developed pneumothorax, of which one (1.9%) needed chest tube drainage. No other adverse events were reported. 

#### 3.2.2. Ion^TM^ Robotic System

The PRCISION-1 study [27] was a prospective single-blinded randomized controlled cadaveric trial using the Ion^TM^ system. The comparative success in lesion localization and puncture was studied using the ultrathin bronchoscope + radial EBUS (UTB-rEBUS) vs. EMN vs. Ion^TM^ robotic bronchoscopy (RB). Six bronchoscopists performed 60 procedures (one each of the above mentioned) on 20 implanted pseudotumors in 5 cadaveric models that were intubated and mechanically ventilated for the procedures. To minimize localization bias, investigators were required to perform UTB-rEBUS before EMN or RB and were prohibited from using rEBUS during the EMN and RB procedures. The mean nodule diameter was 16.5 mm with the CT bronchus sign seen in 50% of them. Nodules were distributed in all lobes with 80% of them intentionally placed in the peripheral 1/3rd. Navigation was considered successful for UTB when rEBUS confirmed visualization and for EMN/RB systems when the virtual pathway confirmed reaching the target. Tool-in-lesion confirmation for biopsy was accomplished using the cone-beam CT for each of these procedures. RB performed significantly better in nodule localization (100% vs. 85% and 65% for EMN and UTB-rEBUS, respectively) as well as needle puncture (80% vs. 45% and 25% for EMN and UTB-rEBUS, respectively). When comparing the needle-to-target “miss” distance among nodules with unsuccessful biopsies, RB again performed better and was found to reach closer to the nodules (0.4 cm vs. 0.7 cm vs. 1.3 cm for RB vs. EMN vs. UTB-rEBUS, respectively).

The first human trial of the Ion^TM^ shape-sensing robotic system by Fielding et al. examined a set of predefined safety and feasibility endpoints [28]. Thirty patients with 30 peripheral nodules were enrolled in this single-center study. Two experienced bronchoscopists performed the procedure on all the patients. The primary safety endpoint was a composite of pneumothorax and excessive bleeding requiring therapeutic intervention. The ability to navigate to and sample the target lesion(s) was the feasibility endpoint. The mean lesion size was 12.2 ± 4.2, 12.3 ± 3.3, and 11.7 ± 4.1 mm in the axial, coronal, and sagittal planes, respectively. Of these, 41.4% of nodules did not have the bronchus sign on the pre-procedure planning CT scan. All lesions were at least 15 mm away from the visceral pleura. Navigation pathways were created automatically by the software based on the pre-procedure thin-slice CT chest. All patients were intubated and mechanically ventilated under general anesthesia. Navigation was accomplished using the real-time video probe visualization as well as the virtual pathway guidance. Mini-probe rEBUS was able to visualize 93% of the nodules before biopsy, which was performed mainly using the flexible 19- to 23-G Auris transbronchial needles along with other conventional biopsy tools. The primary feasibility endpoint of successful navigation followed by biopsy was reported in 96.6% of cases. The mean procedure time was 63.9 ± 24.4 min, and it expectedly lessened as proceduralists gained experience. The subjects were followed for 6 months and the overall diagnostic sensitivity was assessed at 79.3%, further improved to 88% for malignant lesions. Diagnostic specificity for malignant lesions approached 63.6% (range 30.8–89.1%). From a safety standpoint, no adverse events (device-related events, pneumothorax, and excessive bleeding) were reported.

Bajwa et al. reported their findings of a retrospective review of 76 consecutive cases in an abstract at the recently concluded American Thoracic Society’s 2021 annual conference [29]. All 76 patients underwent the Ion^TM^ robotic bronchoscopy-assisted biopsy of the peripheral lung lesions. The median size of the lesions was 1.7 (range, 0.6–7) cm. The median procedure duration was 58 (range, 23–102) minutes. Radial EBUS was used to confirm lesion localization; the view was concentric in 46% (*n* = 35), eccentric in 30% (*n* = 23) and had no visualization in 24% (*n* = 18). The overall reported diagnostic yield was 92% (*n* = 72) of which 59% were malignant. Rather surprisingly, no correlation was found between the rEBUS view and diagnostic yield. The findings reported in this abstract are yet to be published in a peer-reviewed journal.

## 4. Discussion

Of the three key components of successful peripheral lung lesion biopsy using guided bronchoscopy (navigation to the lesion, confirmation of successful navigation, and precise tissue acquisition), current evidence unambiguously establishes the superior navigation capabilities of both the robotic bronchoscopy platforms. The thin caliber, 4-way maneuverability, and endoscopic visibility of robotic scopes ensure their farther reach through smaller airways with difficult turns during peripheral navigation. Both systems have the ability for the scope to be locked in position once the target has been reached, imparting improved stability during sampling. With that said, confirmation of successful navigation remains a challenge. Table 2 provides a comparative summary of the evidence for both the robotic systems. The cadaveric data for both systems were more promising than the human trials, perhaps because CTBD, respiratory variations, and atelectasis do not impact cadavers as much. As with the preexisting EMN bronchoscopy platforms, robotic platforms also plan their pathways based on the pre-procedure CT chest, which makes them vulnerable to the same CT-to-body divergence (CTBD) phenomenon discussed previously. Additionally, almost all centers currently perform robotic bronchoscopy procedures under general anesthesia with patients endotracheally intubated and mechanically ventilated. As was reported in the i-LOCATE trial [15], intraprocedural atelectasis is common during general anesthesia and can be misleading during rEBUS confirmation leading to inaccurate tissue acquisition. Cone-beam CT (CBCT) can offset this shortcoming to a large extent but has limited availability and expertise. Tissue acquisition will likely be improved with the new flexible needles introduced by both the robotic platforms in addition to the existing range of tools. 

One of the concerns is whether the promising data from these initial trials will stand the test of the real-world experience. We know from the NAVIGATE study [4] that the initial euphoria about the excellent diagnostic yield from the existing EMN systems at the time amounted to ~70% yield in real-world data. To their credit, all published robot trials included smaller lesions predominantly located in the outer 1/3rd of the lungs with almost half of them devoid of the bronchus sign, many of which already had failed diagnostic procedures using the existing modalities including the EMN bronchoscopy. Furthermore, even the current experts in robotic bronchoscopy are understandably new to these technologies and have their own learning curve with the systems and their nuances. As our understanding improves with their evolving experience [21], so will likely the procedure outcomes. It is therefore reasonable to expect the diagnostic yield of the robotic platforms to improve beyond the above stated as proceduralists gain more experience and the technology evolves further. The results of the ongoing larger TARGET (ClinicalTrials.gov Identifier: NCT04182815; Monarch^TM^ system) and PRECIsE (ClinicalTrials.gov Identifier: NCT03893539; Ion^TM^ system) studies will hopefully answer some of these questions. 

## Figures and Tables

**Table 1:** Comparison of the technical aspects of the two platforms.

Platform	Monarch^TM^ (Auris Robotics)	Ion^TM^ (Intuitive Surgical)
Bronchoscope system	Telescopic mother-daughter configuration: 6.0 mm outer sheath and 4.2 mm inner scope	Articulating catheter: 3.5 mm outer diameter with a thin 1.8 mm removable visual probe
Working channel	2.1 mm	2.0 mm
Guidance technology	Electromagnetic navigation + peripheral vision	Fiberoptic shape-sensing + peripheral navigation
Contributing technologies	CT, EMN, rEBUS, live views, fluoroscopy	CT, rEBUS, live views, fluoroscopy
Controller	Two joysticks, buttons for irrigation, aspiration etc.	Trackball and scroll wheel
Steering	4-way steering control with 180-degree deflection either way	180-degree articulation either way
Stability	Sheath and scope assembly locks in position during biopsy	Active robotic-controlled catheter locks in position during biopsy
Biopsy tools	Auris needle (currently recalled); other needles, forceps, brushes	Flexision (Intuitive) needle; other needles, forceps, brushes
Caution	Not recommended in those with IEDs (pacemaker, ICD); risk of interference	No such limitations advised

**Table 2:** Comparative summary of evidence for both robotic platforms.

Studies	Lesion Size (mm)	Peripheral Distribution	Bronchus Sign Present	Distance to Pleura	Diagnostic Sensitivity
**Monarch^TM^**					
Cadaveric					
Chen [23] (*n* = 67)	20.4 (9.6–28.3)	100% < 3 cm from pleura	NS	16 ± 10.6 mm (mean)	65/67 (97%)
Human					
Rojas-Solano [24] (*n* = 15)	26.0 (10–63)	80%	100%	6 (0–34) mm (range)	14/15 (93%) *
Chaddha [25] (*n* = 165)	25.0 ± 15.0	71%	63.5%	NS	114–127/165 (69.1–77%) ^#^
Chen [26] (*n* = 54)	23.2 ± 10.8	100%	59.3%	NS	40/54 (74.1%)
**Ion^TM^**					
Cadaveric					
Yarmus [27] (*n* = 20)	16.5 ± 1.5	80%	50%	NS	16/20 (80%)
Human					
Fielding [28] (*n* = 29)	15.3 ± 4.8	100%	58.6%	>15 mm for all lesions	23/29 (79.3%)
Bajwa [29] (*n* = 76)	17.0 (6–70)	NS	NS	NS	70/76 (92%) ^¥^

*n* = number of lesions biopsied (except Chaddha [25] and Bajwa [29], where *n* = number of cases); NS = not specified; peripheral distribution = lesion in outer 1/3 of lung, or inaccessible using white-light and convex probe EBUS bronchoscope (Chen [23]). * Study did not report diagnostic sensitivity, but 14/15 lesions had diagnostic sampling. ^#^ Assuming the biopsy-proven inflammation (*n* = 13) was nondiagnostic vs. diagnostic. ^¥^ This is an ATS abstract.

## Data Availability

Not applicable.
